# Novel Influenza Vaccines: From Research and Development (R&D) Challenges to Regulatory Responses

**DOI:** 10.3390/vaccines11101573

**Published:** 2023-10-09

**Authors:** Xiangchuan He, Tianxiang Zhang, Shitong Huan, Yue Yang

**Affiliations:** 1 School of Pharmaceutical Sciences, Tsinghua University, Beijing 100084, China; hexiangchuan@foxmail.com (X.H.); txzhangsteven@gmail.com (T.Z.); 2 Key Laboratory of Innovative Drug Research and Evaluation, National Medical Products Administration, Beijing 100084, China; 3 Tsinghua-Peking Center for Life Sciences, Beijing 100084, China; 4 China Office, The Bill & Melinda Gates Foundation, Beijing 100084, China

**Keywords:** novel influenza vaccines, research and development (R&D) challenges, regulatory pathways, prior licensing, influenza pandemics

## Abstract

Influenza vaccines faced significant challenges in achieving sufficient protective efficacy and production efficiency in the past. In recent decades, novel influenza vaccines, characterized by efficient and scalable production, advanced platforms, and new adjuvant technologies, have overcome some of these weaknesses and have been widely licensed. Furthermore, researchers are actively pursuing the development of next-generation and universal influenza vaccines to provide comprehensive protection against potential pandemic subtypes or strains. However, new challenges have emerged as these novel vaccines undergo evaluation and authorization. In this review, we primarily outline the critical challenges and advancements in research and development (R&D) and highlight the improvements in regulatory responses for influenza vaccines.

## 1. Introduction

The influenza virus is one of the primary causes of respiratory diseases. The influenza virus mutates through antigenic “drift” and “shift”, leading to seasonal epidemics and even pandemics. According to the World Health Organization (WHO), each year, influenza epidemics lead to severe illness in 3 to 5 million people and cause between 290,000 and 650,000 deaths worldwide [1,2]. Acute complications from influenza primarily impact vulnerable populations, such as young children, the elderly, pregnant women, and individuals with specific chronic medical conditions. This scenario significantly heightens the morbidity and mortality rates associated with influenza infections [3].

Vaccination against influenza is the most effective strategy for preventing influenza-related infections, reducing hospitalization, and even preventing deaths. As a result, the WHO recommends annual vaccination for at-risk populations [4]. Conventional influenza virus vaccines primarily target the virus’s surface glycoprotein known as hemagglutinin (HA). Researchers have identified 18 subtypes of influenza A hemagglutinin (HA), labeled as H1 to H18, and two lineages of influenza B. Conventional seasonal inactivated influenza vaccines typically contain three or four components. These components can be either antigens or strains. Trivalent vaccines include two influenza A subtypes (H1N1 and H3N2) along with one influenza B lineage (Yamagata). Quadrivalent vaccines, on the other hand, consist of two influenza A subtypes (H1N1 and H3N2) and two influenza B lineages (Victoria and Yamagata). These seasonal vaccines primarily induce immune responses targeted at the specific HA subtypes they contain. However, their effectiveness in offering protection against new influenza subtypes is limited, rendering them inadequate for pandemic preparedness [5].

Influenza A viruses are zoonotic, meaning they can infect a wide range of animal species and have the potential to spill over from the animal reservoir, causing human pandemics. Thus, pandemic influenza vaccines are usually stockpiled in response to potential outbreaks. Currently, two types of pandemic influenza vaccines have been authorized: one developed in response to the 2009 influenza pandemic (H1N1) and another targeting avian influenza viruses (H5N1) that have not yet been widely spread in the population [6].

Typically, influenza vaccines utilize three licensed platform technologies: inactivated influenza vaccines (whole, split, and subunit) (IIVs), live attenuated influenza vaccines (LAIVs), and recombinant influenza vaccines (RIVs). These vaccines are manufactured through three primary production technologies: egg-based (most common), cell-based, and live attenuated (weakened) vaccines. Correspondingly, LAIVs and IIVs (whole virus and split) are produced in chicken eggs, IIVs (subunit) are produced in chicken eggs and cells (MDCK), while RIVs are produced in cells (insect cells). In addition, a number of novel influenza vaccines, such as influenza vaccines based on an mRNA platform and universal vaccines with broad protection, are in the clinical phase, and will help strengthen scalable manufacturing and pandemic responses [7,8,9,10,11].

## 2. Influenza Vaccines and Current Challenges

### 2.1. Virus Variation and Vaccine Effectiveness

The WHO Global Influenza Surveillance and Response System (GISRS) constantly monitors influenza viruses circulating worldwide. Each year, it predicts prevalent strains and recommends trivalent/quadrivalent influenza vaccines. Based on recommendations from GISRS, every country has the autonomy to determine the components of their annual influenza vaccines [12]. However, deviations in predictions may lead to a mismatch between the vaccine strains and the real pandemic viruses, and thus, the vaccine effectiveness can decrease to as low as 10%. Even when there is a perfect match with the pandemic strain, the vaccine effectiveness is still capped at a maximum of 60%. In addition, seasonal influenza vaccines are effective for only 6–8 months with low immunogenicity and rapidly diminishing responses. Furthermore, the diverse evolution of pandemic influenza virus strains presents new challenges for vaccine design and pandemic preparedness [13]. These factors collectively contribute to the fluctuating effectiveness and duration of influenza vaccines. Consequently, seasonal influenza vaccines must be regularly updated, often requiring annual vaccination.

### 2.2. Insufficient and Untimely Production

The WHO estimates that around 1.2 billion people worldwide are at a high risk of infection with influenza. However, the current annual global production capacity of the seasonal influenza vaccine is limited to only 350 million doses. This limitation poses a challenge in providing coverage for all high-risk populations in the event of an outbreak. For instance, during the 2009 H1N1 pandemic, many countries encountered difficulties in producing an adequate supply of vaccines promptly, resulting in delayed vaccinations [14].

Currently, most influenza vaccines are produced in chicken embryos (eggs), and this conventional method for influenza vaccine manufacturing has been used for more than 70 years. The main advantages of the egg-based platform include an excellent production capacity and a low production cost that allows global access to the vaccine. However, the egg-based platform presents some inherent limitations, such as the dependence on large numbers of eggs, egg-adaptive mutations, difficulties in automation, and long time frames (6–8 months). These limitations in egg-based production have posed a significant speed-limiting challenge for ensuring timely vaccine supply in response to potential pandemics [15].

### 2.3. Populations’ Adaptation

Existing conventional influenza vaccines are less effective/insufficient for high-risk and immunocompromised populations, resulting in adaptation limitations. In order to improve the immunogenicity, protective efficacy, and production flexibility of influenza vaccines, various iterative strategies targeting at-risk populations are actively developing including new technical routes, new adjuvants, dose enhancement, and process changes [16,17,18].

## 3. Influenza Vaccine Iteration

### 3.1. Novel Technology Platforms and Universal Designs

Over the past century, influenza vaccines have undergone a series of advancements, evolving from monovalent to multivalent formulations. Inactivated split and subunit vaccines (IIV) were successively authorized during the period from the 1930s to the 1980s. Since the beginning of the 21st century, there have been significant breakthroughs in technology platforms and processes for authorized influenza vaccines. For example, in 2002, the first LAIV was approved in the United States; in 2007, the first cell-based subunit IIV was approved in the EU; and in 2013, the first RIV was approved in the United States [19,20].

Some subunit (IIV) and all recombinant influenza vaccines can be produced using cell-based technology. Cell-based production has many advantages over the egg-based method, including production flexibility (no reliance on eggs), reduced mutation rates, easier scale-up manufacturing, faster start up, higher initial purity (less allergy risks), and shorter production cycles (3–4 months). These benefits can facilitate the rapid production and release of vaccines during influenza pandemics [20]. However, given the limited production in early stages, the cost–price ratio of cell-based vaccines is relatively high. Moreover, it is reported that RIVs have more advantages, including nearly 30% higher effectiveness compared to conventional IIVs [15,21].

Currently, in both the Europe and the US, trivalent influenza vaccines have been replaced by quadrivalent options, and subunit IIV and cell-based RIV vaccines have gained widespread usage. Recently, the spotlight has turned to enhanced vaccine strategies based on novel platforms (technologies) and universal designs, including the development of next-generation and universal influenza vaccines. Next-generation influenza vaccines are defined here as novel platforms (technologies) with advantages in performance and/or production, which are based on a platform (technology) licensed for other kinds of vaccines, such as mRNA-based influenza vaccines [22]. The development strategy of universal influenza vaccines is usually defined as a conserved antigen target with novel vaccine platforms. For example, universal influenza vaccines can be designed to conserved regions or epitopes to provide broader protection, and can also be developed based on VLP, nanoparticle, viral vector, and nucleic-acid-based platforms to enhance efficacy and durability. Moreover, with expectations of cross-protective antibodies and/or T-cell responses against different strains or subtypes, universal influenza vaccines are designed to protect from all influenza A and influenza B virus subtypes for long-lasting efficacy (Supplementary Table S1) [23]. These novel influenza vaccines are striving to achieve effective and durable protection, but they may face regulatory hurdles due to antigen- and platform-related efficacy evaluation [24,25].

### 3.2. Novel Adjuvant Vaccines

Adjuvants are substances added to a vaccine to induce stronger protection against vaccine-preventable diseases (VPDs), and can improve the ability of the host immune system to recognize vaccine antigens and respond to them. Conventionally, alum is the most common adjuvant used in influenza vaccines, which often covers aluminum salts, including aluminum phosphate and aluminum hydroxide. Strikingly, the immunological mechanism of action for alum is still not entirely understood. In the last 20 years, novel adjuvants for influenza vaccines have been developed to increase the strength and breadth of immune responses by inducing cross-reactive antibodies against mutated virus strains. For example, two oil-in-water adjuvants, AS03 (mixture of squalene, DL-α-tocopherol, and polysorbate 80) and MF59 (mixture of squalene, polysorbate 80, and sorbitan trioleate), act in a similar fashion by engaging the innate immune system, leading to cellular recruitment and antigen uptake at the site of vaccination [26].

Novel adjuvants AS03 and MF59, rather than conventional aluminum adjuvants, have produced significant improvements in the effectiveness of H1N1 or H5N1 pandemic influenza vaccines. However, during the 2009–2010 H1N1 pandemic, the AS03-adjuvanted H1N1 pandemic vaccine (Pandemrix) was shown to be associated with an increase in cases of narcolepsy in Europe. In 2018, a study analyzed the vaccine safety data from 10 global study sites, and finally claimed no associations between AS03- or MF59-adjuvanted pandemic H1N1 vaccines and narcolepsy [27]. To boost the immune response in the elderly, the new adjuvant MF59 has also been applied to seasonal IIVs [28,29]. These novel adjuvants can play an important role in pandemic preparedness as they can reduce the amount of antigen required in a single dose, increasing the number of doses of vaccine that can be produced, so that a large number of people can be protected as quickly as possible.

### 3.3. Whole Population Coverage

With the immunogenicity challenge, vaccines need to be efficacious particularly in high-risk populations, such as in the elderly. Recent studies showed that high-dose, novel adjuvanted IIVs and RIVs have superior immunogenicity in at-risk populations [30]. Since 2010, the US CDC has recommended annual vaccination with licensed influenza vaccines for populations older than 6 months [31]. The first high-dose inactivated influenza vaccine and first novel adjuvanted influenza vaccine (MF59) specifically for the elderly were authorized in 2009 and in 2015 in the US, respectively. While routine inactivated split and subunit tetravalent influenza vaccines have been expanded to provide whole population coverage (≥6 months) globally, the high-dose, novel adjuvanted influenza vaccines specifically for the elderly have only been widely used in the EU and the US [32].

## 4. Regulatory Pathways and Flexibility in Response to Pandemics

### 4.1. Accelerated Pathways

The Food and Drug Administration (FDA) is the regulatory authority for drug licensing in the US. The Center for Biologics Evaluation and Research (CBER) of the FDA is responsible for reviewing biologics license applications (BLAs) for vaccines and other related biologics. Conventional BLAs should contain extensive preclinical and clinical data to demonstrate the efficacy, safety, purity, and potency of the vaccine, as well as detailed information on the manufacturing processes and facilities [33]. The FDA has established a set of comprehensive review procedures, which are applied to the traditional and accelerated reviews. Accelerated review includes fast-track (FT), breakthrough therapy designation (BTD), priority review (PR), and accelerated approval (AA) [34].

The accelerated approval allows the FDA, based on preliminary evidence, to approve new drugs offering significant benefit for serious medical conditions with unmet needs. The confirmatory evidence can be gathered using post-market requirements or mandated studies [35]. Influenza is a serious and sometimes life-threatening disease. In accordance with 21 CFR 601.41, the FDA allows the licensing of inactivated influenza vaccines through an accelerated approval pathway when there is an insufficient supply of seasonal influenza vaccines for the at-risk populations recommended by the US CDC annually (Table 1) [36].

The European Medicines Agency (EMA) is responsible for the assessment and regulation of medicines in the EU. The Committee for Medicinal Products for Human Use (CHMP) of the EMA reviews and provides scientific advice on licensing new medicinal products, including vaccines, and all member states are expected to adhere to the scientific principles and regulatory guidance from CHMP. The EMA has established several regulatory approval pathways, such as the centralized procedure, decentralized procedure, mutual recognition procedure, and national procedure. The marketing authorization applications (MAA) for influenza vaccines can be submitted through either the centralized or national procedure. According to the Appendix to Regulation (EC) No. 726/2004, the centralized procedure is the compulsory pathway when certain technologies are applied in vaccines (such as recombinant DNA) [37,38]. Besides the standard pathway in the centralized procedure, there are also accelerated pathways including PRIME, accelerated assessment (AA), conditional marketing authorization (CMA), and exceptional circumstances (Table 1) [39].

In addition, the EMA classifies influenza vaccines into three categories, namely seasonal, pre-pandemic, and pandemic preparedness influenza vaccines. Seasonal influenza vaccines are usually readily available and dependent on predictive algorithms and epidemiology. These include trivalent and quadrivalent influenza vaccines immunized annually before influenza recurrence. Pre-pandemic influenza vaccines, also known as zoonotic influenza vaccines, are intended for immunization during outbreaks of virus strains of an animal origin (such as A/H5N1), including when public health authorities anticipate a possible pandemic caused by the virus strain or a similar strain. Pandemic preparedness influenza vaccines (previously termed mock-up vaccines) normally contain a strain of bird flu virus (such as A/H5N1) that few people in the world have already been exposed to and that could potentially cause a pandemic. These influenza vaccines mainly use the accelerated assessment, CMA, and exceptional approval procedures. CMA and exceptional approval play an important role in public health emergencies and pandemics [40,41,42].

The China National Medical Products Administration (NMPA) has established a systematic review procedure. According to the Provisions for Drug Registration (2020-issued), the accelerated pathways in China consist of breakthrough therapy, conditional approval, prioritized review, and special approval procedures for certain conditions [43]. For influenza vaccines, the main procedures are traditional review, priority review, and special approval. For example, the Nasal Spray Lyophilized Live Attenuated Influenza Vaccine (Changchun Baike Ltd., Changchun, China) was approved with priority review procedures, and the pandemic influenza A (H1N1) vaccines (Sinovac Biotech Ltd., Beijing, China, Changchun Institute of Biological Products Co., Ltd., Changchun, China) were approved with special approval procedures [44]. The special approval enables the fast review and approval of preventive vaccines in response to public health emergencies and potential pandemics. The record of the fastest review time is within 24 h (Table 1 and Table 4) [45,46].

**Table 1:** Accelerated pathways for licensing of influenza vaccines.

Regulatory Authority	FDA	EMA	NMPA
Procedure	Accelerated approval	Accelerated assessment	Priority review
Statutory article and guidance	21 CFR 601.41	Reg. (EC) No. 726/2004 Article 14	Provisions for Drug Registration (2020-issued) Chapter 4
Application period	Clinical phase 2/3	Marketing authorization applications	Biologics license applications
Applicable condition	New drugs offering significant benefit for serious medical conditions with unmet needs	Clinically urgent medicines for public health needs	Vaccines urgently needed for disease prevention and control and innovative vaccines
Accelerating mechanisms/review timelines	Licensed based on preliminary evidence, confirmatory evidence gathered using post-market studies	150 days	130 days

### 4.2. Annual Update Procedures

To change strains (with the same subtype substitution) in seasonal influenza vaccines, the US FDA requires the information on the transmission history and antigenic characterization of the virus seeds. Additionally, strain change supplement applications for seasonal influenza vaccines should be submitted annually in accordance with 21 CFR 601.2. For IIV and RIV, additional clinical data specific to the new strain were not required, whereas for LAIV, clinical data of approximately 300 adults to verify adequate attenuation were required in the supplement application (Table 2) [47,48,49].

During the influenza season, the strains of seasonal influenza vaccines approved through a centralized procedure can be annually updated in the EU, as outlined in Article 18 of Regulation (EC) No. 1234/2008. The annual update procedure does not require pre-market clinical trial data, but only quality-related data for the new strain (same subtype substitution) in the supplement application (Table 2) [49]. The China NMPA has not disclosed annual update procedures and relevant guidance.

**Table 2:** Annual update procedures for seasonal influenza vaccines.

Regulatory Authority	FDA	EMA
Procedures	Supplement application	Strain change supplement application
Statutory articles and guidance	21 CFR 601.2	Reg. (EC) No. 1234/2008 Article 18
Supplemental data requirement	♦ For IIV and RIV, no new clinical data are required. ♦ For LAIV, new clinical data of approximately 300 adults are required.	♦ For same-subtype substitutions, no pre-market clinical data, but quality-related data for the new strain, are required.

### 4.3. Prior Licensing and Strain Changes’ Procedures

Prior licensing procedures allow influenza vaccines to be developed in advance of a pandemic, and in some cases, influenza vaccines can be approved but not authorized prior to a pandemic. Typically, the prior licensing requires the vaccine candidates to have the same production process and specifications as existing influenza vaccines, especially antigen contents, excipients, and adjuvant systems. Such vaccines normally contain a strain of bird flu virus that few people in the world have already been exposed to and that could potentially cause a pandemic. In the event of a pandemic, once the virus strain causing the pandemic is identified, the manufacturer can include this strain in the prior licensed vaccine for a closely matching subtype (strain) and apply for the vaccine to be authorized as a final pandemic vaccine. The authorization of the final pandemic vaccine can be relatively fast as authorities have already assessed the vaccine safety and efficacy with other potential pandemic strains [48,50].

The FDA allows a regulatory pathway facilitated by prior licensing of a vaccine consisting of a novel pandemic subtype referred to as the prototype. The novel pandemic influenza virus (prototype) would receive licensing before the occurrence of a pandemic. This is performed to ensure that, if a pandemic occurs, a candidate vaccine virus that is a close match could be introduced as a supplemental strain change to the existing license. In such circumstances, clinical trials were conducted to establish the safety and immunogenicity of the chosen dosage and schedule. The effectiveness of vaccines was inferred from the established efficacy of the seasonal vaccine produced by the same manufacturer using the same process [50].

To date, the FDA has licensed four pandemic H1N1 influenza vaccines. All of them were approved with an annual supplement application based on the authorized trivalent seasonal influenza vaccines, in which the matched virus subtype (H1N1 is not novel) is included in candidate vaccines. In this context, the clinical trial was not required to determine the vaccination dosages and schedules for the new strain. However, for new subtypes, such as H5N1 and H7N9, clinical trials on the dosage, schedule, and safety of new strains were required. Such novel subtypes of vaccines are recommended to be licensed prior to a pandemic. Once in pandemics, the most closely matched licensed vaccine strains can be updated to pandemic vaccines under a procedure similar to the seasonal vaccine annual supplement application (Table 3). In addition, the FDA has licensed three pandemic H5N1 influenza vaccines, including the Influenza Virus Vaccine H5N1 (National Stockpile), the Influenza A (H5N1) Virus Monovalent Vaccine (AS03), and the AUDENZ (MF59). The AUDENZ (MF59) was approved using accelerated approval with immunological surrogate endpoints and a commitment was made to conduct clinical efficacy studies once there is a pandemic [51,52].

The EMA has established marketing authorization procedures for pandemic preparedness vaccines prior to pandemics. It is generally accepted that the immunogenicity and safety data of a pandemic vaccine can be predicted from the data of an approved pandemic preparedness vaccine. In accordance with Article 21 of Regulation 1234/2008/EC, in a pandemic, the marketing authorization holder (MAH) should submit a vaccine strain change application to replace the pandemic preparedness strain with the pandemic strain. Different from same-subtype substitutions with only quality data, for novel subtype substitutions, e.g., H7N9 replacing H5N1, additional non-clinical quality and clinical data are required, and case-by-case communications may be needed (Table 3) [53,54].

The EMA has licensed five H1N1 pandemic preparedness vaccines. Among them, three were converted from H1N1 pre-pandemic vaccines in 2009 by changing the supplement procedure of a pandemic strain, namely Focetria, Pandemrix, and Celvapan. Two other pandemic vaccines, namely Arepanrix and Humenza, were licensed with an emergency procedure in 2010. In addition, the EMA has licensed four H5N1 pandemic preparedness vaccines, including Foclivia, Adjupanrix, Pandemic Influenza Vaccine H5N1 Baxter AG, and Pandemic Influenza Vaccine H5N1 AstraZeneca, which could be converted to a pandemic influenza vaccine during a pandemic [55,56]. The China NMPA has not disclosed any approval procedures for pre-pandemic vaccines or relevant guidance.

**Table 3:** Prior licensing and strains’ change procedures for pandemic vaccines.

Regulatory Authority	FDA	EMA
Statutory articles and guidance	Guidance for industry: Clinical data needed to support the licensing of pandemic influenza vaccines	Article 21 of Regulation 1234/2008/EC
Procedure 1		
Same subtypes	Supplement application	
Data requirement	Same as annual supplement for seasonal influenza vaccines	
Procedure 2		
Novel subtypes	Traditional pathway + supplement	Pre-pandemic procedures + supplement
	Accelerated approval + supplement	
Data requirement	Clinical trials for safety and immunogenicity of the selected dose and schedule	Non-clinical quality and clinical data; case-by-case communications may be needed

### 4.4. Emergency Procedures and Authorization

Emergency use procedures and authorization present another route for review and market access. In China, the Vaccine Administration Law specifically addresses the emergency use of vaccines (biological products for prophylactic use), with Article 20 (2) stipulating that “in the event of a particularly serious public health emergency or other emergency that poses a serious threat to public health, the health department of the State Council should make a proposal for the emergency use of vaccines in accordance with the needs for the prevention and control of infectious diseases”. The drug supervision and management department of the State Council may, after demonstration and consent, use the vaccine within a certain range and period of time. However, China has not yet established a comprehensive and enforceable authorization system for the emergency use of pharmaceutical products (Table 4) [43].

In the US, under Section 564 of the FD&C Act, the FDA may issue an Emergency Use Authorization (EUA) to allow the distribution of unapproved drugs or vaccines for the diagnosis, prevention, or treatment of serious or life-threatening illnesses during a public health emergency when there are no adequate, approved, and available alternatives. The application for an EUA must be supported with data from clinical trials demonstrating safety and efficacy. In the case of vaccines, it should also include immunogenicity data and adverse event monitoring and reporting, and the review timeline will be compressed to months or even weeks. Once the public health emergency is over, the EUA is no longer valid, at which point formal approval must be obtained to continue using the product for the same indications as its EUA (Table 4) [57,58].

In the EU, making products available as quickly as possible in an emergency situation relies on conditional marketing authorization (CMA) or emergency authorization procedures in member states. In pandemics, emergency procedures allow for rapid approval of new pandemic influenza vaccines, which are approved more quickly (70 days) compared to standard procedures (210 days). However, the emergency procedure (70 days) is much slower than the above strain change procedure for pre-pandemic vaccines (15–25 days). Currently pre-pandemic vaccines (mock-up vaccines) are the most rapid procedure for approving H1N1 vaccines in the EU (Table 4) [59].

In April 2023, the European Commission, in its proposal for amendments to the general medicine legislation (Article 3 (2) of the draft directive), proposed the introduction of a Temporary Emergency Marketing Authorization (TEMA) in the event of “public health emergencies” as a “flexible, rapid and simplified” process for the temporary authorization of the use and distribution of unregulated vaccines with a view to more rapidly authorize the use of medicines in any future public health emergency. In addition, according to Article 23 of Regulation (EU) 2022/2371, the commission will only grant a TEMA if there is a recognized and ongoing “public health emergency” at the EU level [60].

**Table 4:** Emergency regulatory pathways for influenza vaccines’ authorization.

Regulatory Authority	FDA	EMA	NMPA	
Names	Emergency Use Authorization	Conditional Marketing Authorization	Emergency Authorization	Special Approval
Statutory articles and guidance	FD&CA 564	Reg. (EC) No. 726/2004 Article 14 (AA) Reg. (EC) No. 507/2006	Vaccine Administration Law Article 20	Procedures for Drugs Special Approval
Regulatory pathways	Emergency authorization (not formal approval)	Approval procedure	Emergency authorization (not formal approval)	Approval procedure
Applicable condition	♦ Serious or life-threatening disease or condition ♦ Evidence of effectiveness ♦ Risk-benefit Analysis ♦ No alternatives	♦ Fulfil an unmet medical need ♦ Positive benefit-risk balance	♦ Serious public health emergency ♦ Other serious threat to public health	♦ State/region emergency ♦ Emergency response to public health emergencies ♦ Proposal for special approval ♦ Others
Review time limit	Hours to days	70 days (emergency procedures) 15–25 days (strain change procedures of mock-up vaccines)	-	Within 15 days
Expiration time	Once public health emergency is over, the EUA is no longer valid, and vaccine still needs formal approval	Valid for 1 year, renewed annually for comprehensive data to convert to formal approval	-	-

## 5. Clinical Evaluation of Influenza Vaccines

In the past, licensed influenza vaccines in Europe and the US, such as trivalent inactivated influenza vaccines and live attenuated vaccines, were approved based on the protective efficacy data. With knowledge accumulation from influenza vaccine research and development, as for inactivated influenza vaccines, hemagglutination inhibition (HI) antibodies have been recognized as a key immunological surrogate endpoint marker for predicting protection [61]. In 1997, the EMA CHMP proposed influenza vaccine guidance to establish the absolute criteria for surrogate endpoints of an HI antibody, including a GMT fold increase, seroconversion rate, and seroprotection rate—the universal standard that has been well referenced and applied by regulatory authorities globally (Table 5) [62,63].

For the iterative development of quadrivalent inactivated seasonal influenza vaccines, regulatory authorities such as the FDA and EMA primarily accepted protective efficacy studies as pivotal results for approval. If applicants applied immunological surrogate endpoints as the supporting evidence of vaccine efficacy for an immune bridging or non-inferiority study, as the supporting evidence of vaccine efficacy, the post-marketing protective efficacy study should be supplemented in accordance with the requirements of the regulatory authorities [48,53].

In early years, trivalent influenza vaccines had been imported into China with using protective efficacy data from overseas. Subsequently, trivalent inactivated influenza vaccine developed by Chinese manufacturers were gradually tested in clinical trials, in which the imported trivalent influenza vaccines with the same strains were usually used as positive controls in non-inferiority comparisons. Moreover, the clinical trial was designed to bridge the efficacy data of imported vaccines through the immunological surrogate endpoint results. In recent years, with the iterative development of a quadrivalent inactivated influenza vaccine by Chinese manufacturers, two licensed trivalent vaccines of covering virus strains from a quadrivalent candidate are usually used as positive controls, and immunological surrogate endpoints are applied to predict efficacy in non-inferiority clinical trials. Furthermore, influenza vaccine evaluation criteria in China are also based on the EMA and FDA standards [63].

At present, new methods for assessing the effectiveness of the next-generation influenza vaccines are actively being studied. The recent EMA guidance no longer relies on serological criteria with predefined protection thresholds to determine the vaccine benefit, but rather on allowing for a more diversified approach to measuring, characterizing, and reporting on influenza vaccine immune responses [39]. In addition, a more comprehensive assessment of influenza-vaccine-induced immune responses is also encouraged in the influenza vaccine guidance released from the NMPA [63].

**Table 5:** Evaluation criteria of influenza vaccine immunogenicity (HI antibody).

Evaluation Criteria of Immunological Endpoint in Clinical Trials		
1. Absolute criteria of surrogate endpoint in placebo-controlled clinical trials		
Eligible people	Adults	The elderly
GMT fold increase	2.5	2
Seroconversion rate	40%	30%
Seroprotection rate	70%	60%
2. Relative criteria of common primary endpoints in non-inferiority trials		
GMT	GMT ratio, 95% CI ≥ 2/3	
Seroconversion	Value difference, 95% CI ≥ −10%	

Definitions of the elderly are people of 60 (Europe and China) or 65 (US) years of age and older.

## 6. Tools and Methodologies for Development and Evaluation of Influenza Vaccines

In recent years, with the great progress in influenza vaccines using novel technology platforms and universal designs, the challenges of evaluating novel influenza vaccines have become increasingly prominent. Throughout the lifecycle of influenza vaccine development, efforts are being made to establish new tools and methodologies that can be adaptively applied to assess the safety, efficacy, and quality of novel influenza vaccines. These efforts include the establishment of product profiles, the identification of immunoprotection on novel technology platforms, and the development of human challenge models for predicting safety and efficacy. With the accumulation of new knowledge and experience, a scientific and rational approach can be established for influenza vaccine evaluation [64,65,66].

### 6.1. Product Profile and Characteristics

The Target Product Profile (TPP) document from WHO outlines the required characteristics of a product targeted for a specific disease, defining the product’s intended use, target population, breadth and durability of protection, and other required attributes, including safety- and efficacy-related properties, as well as preferred and minimum acceptable limits for relevant criteria [66]. If WHO has identified a priority need for a product category, but the development stage is still early, a Preferred Product Characterization (PPC) may be provided, which specifies the WHO preferences in general, but does not specify the minimum acceptable standards. Both the TPP and PPC can be considered as important tools to guide vaccine research and development (R&D) and documentation submissions [67].

WHO released the Next-Generation Improved and Universal Influenza Vaccine PPC document in 2017 to improve influenza vaccines, develop new vaccines, and advocate for influenza vaccines that provide long-lasting protection. However, this universal influenza vaccine is only available against influenza A viruses (Table 6). Additionally, the National Institute of Allergy and Infectious Diseases (NIAID) also concludes that a “universal” vaccine implies at least 75 percent effectiveness in protecting all age groups for a minimum of 1 year against all strains of influenza A, and ideally, working against influenza B with protection lasting for 3 to 5 years [68,69].

WHO and the Bill and Melinda Gates Foundation established the Universal Influenza Vaccine TPP document in 2017, and defined vaccines that can prevent morbidity and mortality caused by all pandemic and emerging influenza A and influenza B virus subtypes. The vaccine is indicated for populations older than 6 weeks and those at a high risk (pregnant women, children, and the elderly), and has a duration of protection of at least 3 to 5 years. It is anticipated that this universal influenza vaccine will address the threat of seasonal and pandemic influenza, thereby alleviating the need for annual seasonal influenza vaccination (Table 6) [70]. In 2018, the National Academy of Medicine (NAM) also concluded that a universal vaccine would be able to counteract influenza B viruses and provide protection for 3 to 5 years. Additionally, the 2019–2030 Global Influenza Strategy, released by WHO in 2019, actively calls for the development of improved, novel, and universal influenza vaccines [71]. According to the strategy, multiple universal-vaccine programs have demonstrated promising preclinical and clinical data [70].

**Table 6:** TPP and PPC characteristics for universal influenza vaccines.

Profile Name	TPP	PPC
Organization	WHO/Bill and Melinda Gates Foundation	WHO
Disease	Universal influenza	Universal-type A influenza
Intended use/Indication	Protection from all circulating and emerging influenza A subtypes and influenza B lineage viruses	Protection against severe influenza A virus illness
Target population	♦ From 6 weeks with no upper age limit ♦ High-risk populations	
Efficacy	♦ Minimum efficacy: 70% ♦ Optimistic efficacy: 85%	♦ Better than currently prequalified non-replicating non-adjuvanted seasonal influenza vaccines
Duration of protection	3–5 years	At least 5 years
Onset of immunity	4 weeks	-
Herd protection	Desirable	-
Dose schedule	♦ Naïve subjects No more than 3 doses over 5 years ♦ Primed subjects Single or two-dose primary series, booster every 5 years	-
Safety	No worse than EPI vaccines	♦ An increase in mild reactogenicity may be acceptable if it prevents severe illness ♦ Severe reactogenicity (no worse than currently prequalified non-replicating non-adjuvanted seasonal influenza vaccines)
Stability/ Shelf life	Minimum 2 years at 2–8 deg C	-
Product registration path	♦ Likely licensed as seasonal vaccine that is also useful for pandemics	-
WHO PQ date	2027	No timeline for prequalification
Primary target delivery channel	♦ Routine immunization ♦ Pre-pandemic global campaign	-
Target counties	All	Low- and middle-income countries

### 6.2. The Correlations of Immunoprotection

Novel influenza vaccines with broad protection, such as universal and whole-population coverage, have triggered the new regulatory challenge of efficacy-evaluating methods that can be adapted to influenza vaccines with different characteristics. The mechanism of novel technology platforms and universal influenza vaccines is not based on the induction of antibodies against the HA receptor-binding domain, but against the more conserved epitopes, such as the NPs, M1 and M2e, NA, and the conserved regions within the hemagglutinin stem domain (HA2) and the receptor-binding domain (HA1) [72].

Hemagglutination (HA) inhibition (HI) antibodies’ titer is an immunological surrogate endpoint of efficacy for inactivated influenza vaccines tested in human challenge studies; however, this measurement only represents the amount of specific antibodies against the structural domains of the HA head. Once the components of a universal influenza vaccine with non-HA epitopes are introduced, protection will not correlate with the HI antibody response [73,74]. The single radial immunodiffusion (SRID) method, which detects antibodies by measuring the hemolytic loop caused by the activation of the complement system, measures all serum antibodies against influenza surface antigens, but is unable to recognize local mucosal or cellular immune responses, such as immunization strategies against the M1 or NP proteins, as well as immunization against live attenuated influenza vaccines [75,76].

Additionally, studies have also demonstrated the protective role of anti-neuraminidase (NA) antibodies. However, the importance of anti-NA antibodies’ titers as an immunological correlate of protection was only supported with data from H1N1 human challenge trials. Data about anti-NA antibody responses in naturally occurring human influenza are limited and have not been well studied [77,78,79].

Although in traditional approval procedure, the indicators for evaluating influenza vaccine efficacy primarily include serum antibody levels, seroconversion rate, laboratory-confirmed influenza, acute respiratory illness or influenza-like illness (ILI) visits, and influenza- and pneumonia-related hospitalizations or deaths, vaccine efficacy is not typically determined with the indicators that correlate with protection. However, the regulatory agencies are encouraging a progressive standardization and validation of new assays before the pivotal studies, and by analyzing potential protective data, one or more immunologic endpoint-related indicators would be tested in pivotal efficacy trials. Through establishing immune correlates of protection with clinical outcomes in efficacy trials, immunologic surrogate endpoints that are “reasonably likely” to predict vaccine efficacy can be ideally identified [39].

### 6.3. Appropriateness of Effectiveness Evaluation among Populations

Currently, placebo-controlled efficacy studies in populations where influenza vaccination is recommended may involve ethical challenges. For example, in the United States, annual vaccination against seasonal influenza is recommended for all persons aged ≥ 6 months except when contraindicated [80]. Infants, young children (6–59 months), and the elderly (65 years or older) are not eligible for inclusion in placebo-controlled efficacy studies. The vaccine is generally evaluated in immunobridging studies based on immunological surrogate endpoints. However, the HI antibody as a surrogate endpoint is primarily based on knowledge from healthy adults vaccinated with H3N2 influenza vaccine in the human challenge studies. It is not known whether the correlations established in healthy adults are transferable to vulnerable children, the elderly, and populations with potential complications [81].

Recent studies have concluded that immune-related protection in the elderly population is primarily related to cell-mediated immunity (CMI rather than humoral-mediated immunity (antibodies) [82]. Therefore, there are limitations to the applicability of HI antibodies in the elderly population. Nonetheless, vaccine assessments have relied on universal standards for decades, yet the reliability challenges are increasingly encountered in practice. In addition, due to the lack of evidence that influenza vaccines induce protective immune responses and immune memory in children, both the EMA and NMPA have recommended protective efficacy studies as the primary evidence for age-expanded development in 6–35-month populations [39,83].

### 6.4. Human Challenge Trials

Human challenge trials (HCTs), also known as controlled human infection modelling (CHIM), simulate interactions between human hosts and pathogens by intentionally exposing carefully selected volunteers to a well-characterized pathogen or a representative surrogate challenge agent under controlled conditions. These trials are used to assess the effectiveness of vaccines against a wide variety of at least 30 different pathogens. More than 15,000 individuals have participated in these trials, which have covered typhoid, cholera, yellow fever, influenza, SARS-CoV-2, and many other diseases [84,85].

In 2016, WHO issued regulatory considerations on vaccine HCTs [85]. In the same year, cholera vaccine CVD 103-HgR (VaxChora, Emergent BioSolutions), supported with positive results from 10-day and 90-day HCTs and data from two safety and immunogenicity trials in healthy adults, became the first vaccine approved by the FDA based on HCT results. The approval can be considered as an ideal use-case for HCTs in vaccine development [86,87,88]. At present, regulatory agencies such as the FDA and EMA accept HCT data as evidence for proof-of-concept, dose determination, and efficacy studies. Currently, due to ethical and cultural differences and other reasons, the NMPA and related agencies in China have not yet allowed the implementation of HCT studies.

HCTs are nonetheless ethically sensitive and raise important questions for healthy volunteers, including (i) the acceptance of intentional infection; (ii) the kinds and levels of benefits; (iii) the acceptable limit of burdens (risks); (iv) the need for protection of third-parties from infection (by participants); (v) fair participant selection/exclusion, (vi) appropriate financial payment of participants; (vii) the potential need for special ethical principles and/or review procedures (e.g., special committees); etc. [89]. In 2020, WHO also published the Key Guidance for the Ethical Acceptability of Human Challenge Studies for New Coronaviruses. It set out eight criteria for HCTs, including scientific rationale, assessment of risks and potential benefits, consultation and participation, coordinated research, site selection, participant selection, expert review, and informed consent, to support the use of an HCT approach to COVID-19 vaccine development [90]. In 2020, the UK was the first country to announce and implement an HCT program for COVID-19 vaccines [91].

HCTs have also played a crucial role in influenza vaccine efficacy studies, but their implementation requires careful consideration of factors such as the challenge dose, administration route, screening assays for pre-existing immunity, age group, and time interval between vaccination and challenge. For instance, in a phase 2 influenza vaccine HCT, a trivalent LAIV vaccine demonstrated a vaccine efficacy (VE) of 85%. In this trial, 60 participants were screened in advance for serum HI antibody titers of 1:8 or lower against vaccine strains [92]. However, in the subsequent phase 3 trial, where pre-screening antibody tests were not conducted, and different endpoints were used, the VE dropped to 9.6% [93]. Moreover, post-marketing studies have reported 19% to 20% effectiveness for pneumonia or influenza endpoints [94]. Nevertheless, a meta-analysis of five studies involving children aged 6 months to 7 years (with comparatively lower pre-existing immunity) showed a combined VE of 83%, aligning with the results from HCT [95]. This suggests the presence of “original antigenic sin” (OAS), a phenomenon that after a second exposure to a different antigen variant of the same virus, the immune system responds with antibodies of reduced intensity and specificity, which can impact the vaccine responses due to pre-existing immunity [96]. Thus, human challenge trials (HCTs) have already demonstrated their value in establishing early proofs of concept for vaccine efficacy in humans, guiding vaccine selection and addressing critical knowledge gaps related to transmission, pathogenesis, and immune protection [97].

Furthermore, well-designed human challenge trials could support novel influenza vaccines. They can demonstrate the breadth of protection by exposing individuals to new strains derived from vaccine strains and explore correlates of protection for advanced technology platforms [98]. Moreover, during a global pandemic, aside from the standard initial safety assessments, vaccine dose determination, and immunogenicity studies (CHIM phases 1/2), human challenge trials (HCTs) could offer a viable means to bypass phase 3 testing and expedite the approval of effective and high-priority vaccines [99].

## 7. Conclusions

Novel influenza vaccines with advanced technologies have significantly improved the effectiveness and efficiency of vaccination. Moreover, next-generation mRNA-based and universal influenza vaccines with broad protection are currently in advanced stages of clinical trials, and are likely to be approved within the next few years. To address the challenge of novel vaccines’ evaluation, adaptive tools and methodologies have been gradually explored from scientific research to regulatory application. Furthermore, regulatory pathways for influenza vaccines with accelerated and flexible procedures have been established to allow for timely responses to pandemics. In the future, these collective efforts are expected to support the authorization of novel influenza vaccines and preparedness for influenza outbreaks.

## Data Availability

No new data were created or analyzed in this study. Data sharing is not applicable to this article.

## Supplementary Materials

The following supporting information can be downloaded at: https://www.mdpi.com/article/10.3390/vaccines11101573/s1, Table S1: The classifications and characteristics of influenza vaccines referred in this review.
