# Supplementary material for: Novel Influenza Vaccines: From Research and Development (R&D) Challenges to Regulatory Responses
Source: Vaccines (Basel). 2023 Oct 9;11(10):1573. doi: 10.3390/vaccines11101573 (PMC10610648; doi:10.3390/vaccines11101573)
Supplement: Supplementary file 1 [file vaccines-11-01573-s001.zip › vaccines-2607534-supplementary.pdf]

Supplementary Table S1. The classifications and characteristics of influenza vaccines referred in this review

<table>
<tr><th colspan="2">Classification</th><th>Definition</th><th>Composition /Indication</th><th>Platform / production technology / design strategy</th><th>Approval conditions</th></tr>
<tr><td colspan="2">Conventional vaccines</td><td>Platforms (technologies) in influenza vaccines licensed before 21st century</td><td rowspan="3">H5N1; H1N1; H3N2; Yamagata; Victoria</td><td>Platform: split (IIV) , subunit (IIV); Production technology: egg-based</td><td rowspan="2">Y</td></tr>
<tr><td rowspan="4">Novel vaccines</td><td>Current iterative vaccines</td><td>Platforms (technologies) in influenza vaccines licensed after 21st century</td><td>Platform: LAIV, RIV, new adjuvants; Production technology: cell-based production</td></tr>
<tr><td>Next-generation vaccines</td><td>Novel platforms (technologies) licensed for other vaccines</td><td>Platform: mRNA, viral vector platform etc.</td><td>N</td></tr>
<tr><td rowspan="2">Universal (design) vaccines</td><td rowspan="2">Prevent morbidity and mortality caused by all pandemic and emerging influenza A and influenza B viruse subtypes</td><td rowspan="2">Protection from all influenza A and influenza B viruse subtypes and long-lasting efficacy (3-5 years)</td><td>Platform: VLP, nanoparticle, viral vector and nucliec acid etc.</td><td rowspan="2">N</td></tr>
<tr><td>Design strategy: conserved antigena such as HA stalk, M2e, NP, NA etc.</td></tr>
</table>
